# First person – Eric Clark

**DOI:** 10.1242/dmm.043901

**Published:** 2020-01-28

## Abstract

First Person is a series of interviews with the first authors of a selection of papers published in Disease Models & Mechanisms, helping early-career researchers promote themselves alongside their papers. Eric Clark is first author on ‘Establishment and validation of an endoplasmic reticulum stress reporter to monitor zebrafish ATF6 activity in development and disease’, published in DMM. Eric is a PhD student in the lab of Brian Link at the Medical College of Wisconsin, Milwaukee, WI, USA, investigating cellular stress pathways involved in neurodegeneration.


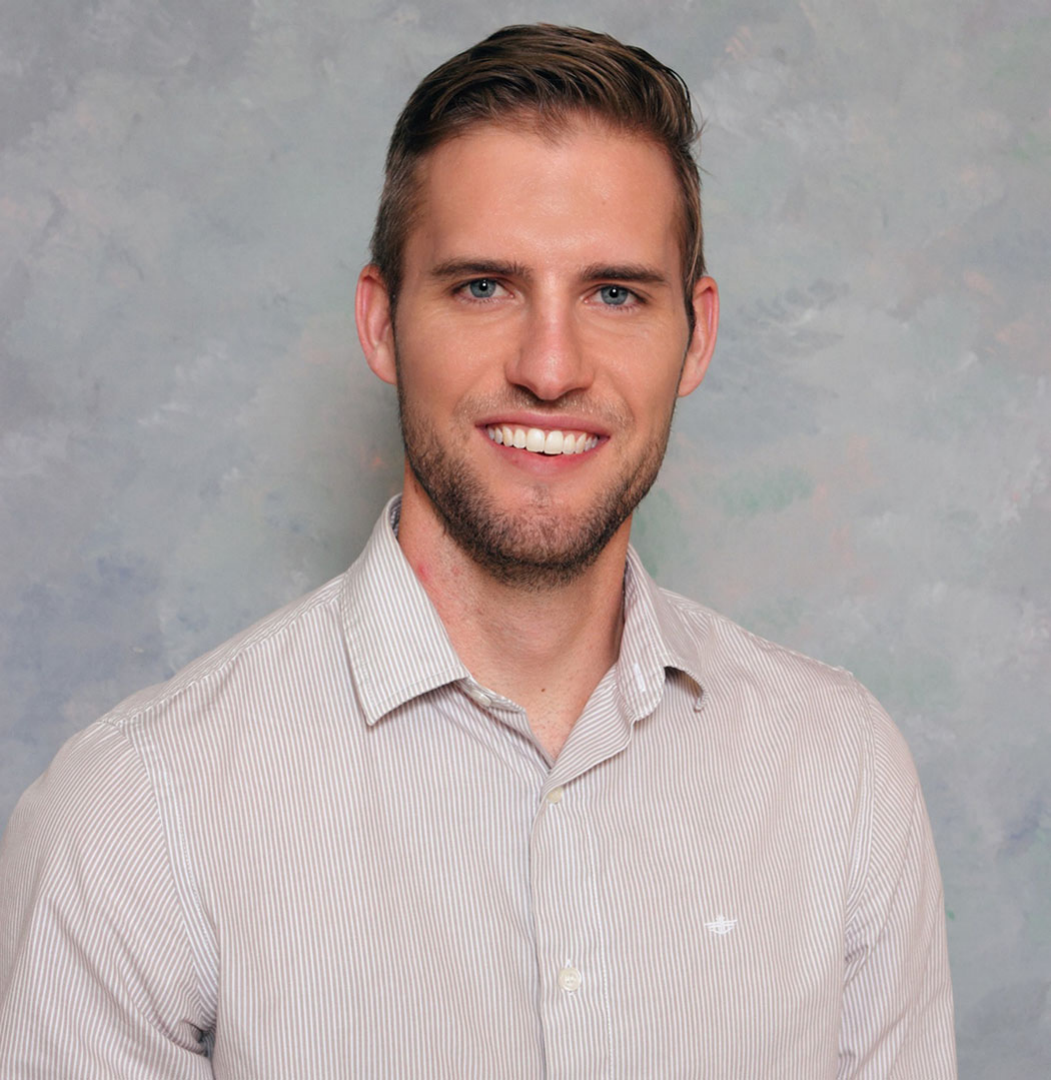


**Eric Clark**

**How would you explain the main findings of your paper to non-scientific family and friends?**

Endoplasmic reticulum (ER) stress is a pathway activated in multiple neurodegenerative diseases. We created a zebrafish line that can monitor activity of a specific protein within the ER stress system called ATF6. This zebrafish line was used to characterize ER stress during development and in disease states including amyotrophic lateral sclerosis (ALS) and retinal cone photoreceptor dystrophy.

“The reporter that we developed is specific for the ATF6 branch of the ER stress pathway and is the first *in vivo* reporter to monitor ATF6 activity.”

**What are the potential implications of these results for your field of research?**

The reporter that we developed is specific for the ATF6 branch of the ER stress pathway and is the first *in vivo* reporter to monitor ATF6 activity. Therefore, this research tool should advance our understanding of ATF6 signaling during development and disease processes. Specifically, the transgenic line could be used for chemical or genetic screens to provide a whole-animal readout for ATF6 activity. ATF6-responding cells can be sorted based on fluorescence and investigated using transcriptomics, metabolomics or proteomics to reveal susceptible cell types and cell states. In addition, the reporter can be coupled with other ER stress reporters to understand how different branches of the ER stress pathway are coordinated spatially and temporally with different biological processes, including normal homeostasis and disease states.

**What are the main advantages and drawbacks of the model system you have used as it relates to the disease you are investigating?**

Zebrafish are transparent during developmental stages. This allows us to watch stress pathway activation dynamically and across multiple tissue types. Zebrafish and human genes are commonly conserved, and genes that cause human disease can be investigated using the zebrafish model system. Unfortunately, zebrafish do not stay transparent into adulthood; however, pigment mutants exist that reduce opacity in adults. In addition, fixed adult zebrafish tissues can be dissected and cleared to image fluorescent reporters after transparency is lost. This is what was done in the featured image where the brain was dissected and cleared, and neurons were imaged throughout the brain.

**What has surprised you the most while conducting your research?**

In our experiments overexpressing ATF6 in zebrafish we noticed that there was an increase in *ATF6* reporter activity in the same cells, suggesting an expected autonomous effect. However, we were surprised to see activation of the *ATF6* reporter in cells proximal to ones overexpressing ATF6, suggesting a non-autonomous effect. Other recent publications have mentioned non-autonomous activation of another ER stress pathway protein, XBP1. We believe that investigating non-autonomous activation of ATF6 could be an interesting future direction.

**Describe what you think is the most significant challenge impacting your research at this time and how will this be addressed over the next 10 years?**

Often in research a complicated disease is broken down to study one aspect at a time, which is necessary to understand that one component. However, neurodegenerative diseases are multifactorial with activation of many stress pathways in a cell at once. Furthermore, multiple cells, tissues and even organ systems can contribute to the disease phenotype. One challenge is to identify individual mechanisms that malfunction at the beginning of disease onset and treating the mechanism in the context of other complicated factors of the disease. When using the zebrafish model, our reporter can be investigated with other fluorescent reporters to understand activation of stress pathway networks throughout zebrafish tissues over time. My hope is that studies in zebrafish using the *ATF6* reporter can provide data to develop biomarkers, therapeutics and an overall better understanding of complex disease progression in humans.

**Figure:**
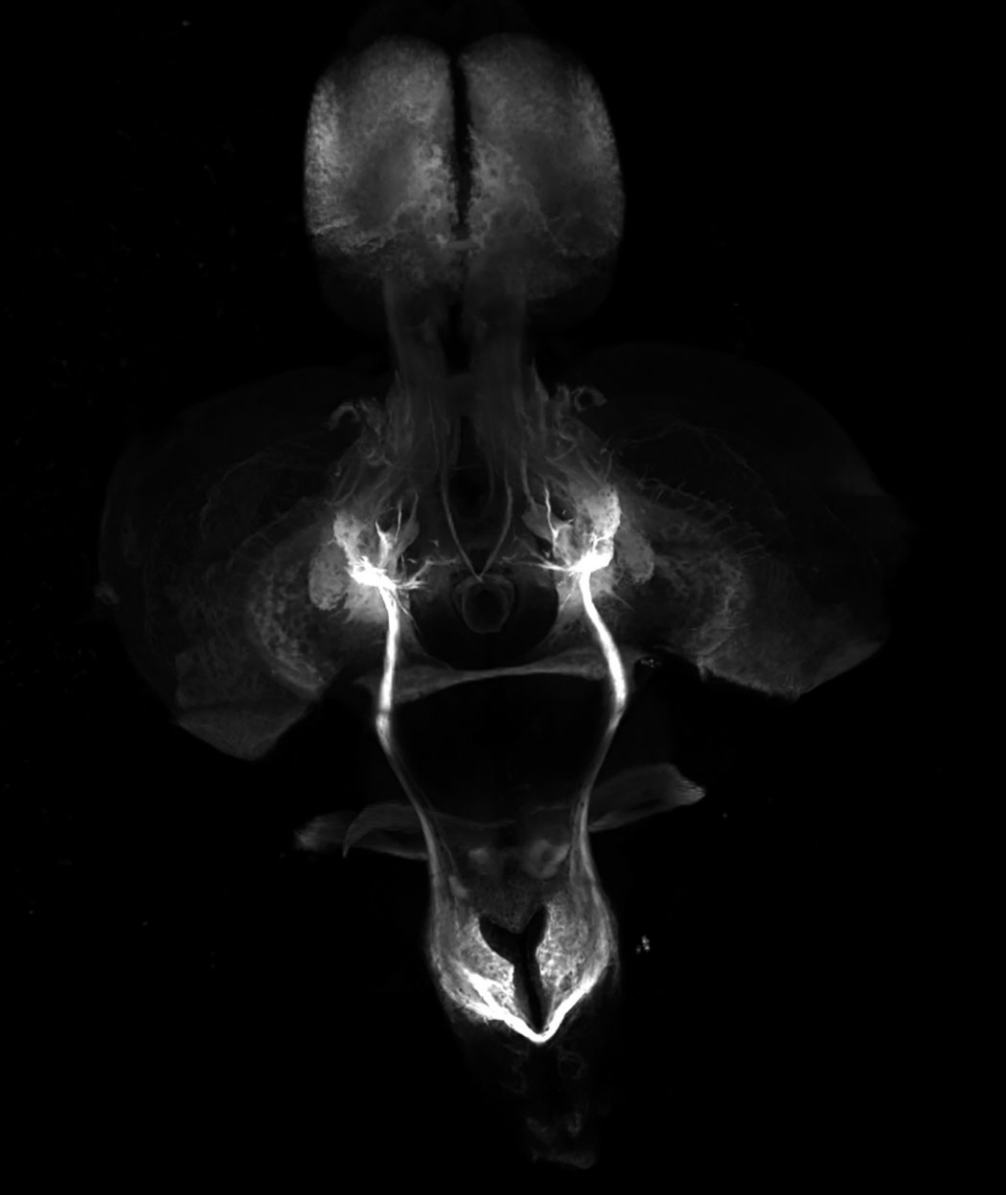
**Passive-clarity-cleared zebrafish brain expressing a *gap43:*GFP reporter highlighting all neurons in the brain. The image was taken on a Leica SP8 AOBS system using a 488** **nm argon laser line.**

“[…] early-career scientists need independence to develop and test research questions of interest.”

**What changes do you think could improve the professional lives of early-career scientists?**

I think that early-career scientists need independence to develop and test research questions of interest. I have been fortunate in the Link lab to have the freedom to develop resources like the *ATF6* reporter and use them to explore research questions that were interesting to me, and to produce results that can contribute to the scientific community. Continued funding resources for early-career scientists help facilitate this research autonomy as well.

**What's next for you?**

I am currently working on publishing work detailing novel factors in neurodegeneration, in which the *ATF6* reporter has been useful. I will be defending my dissertation soon and am interested in continuing investigating mechanisms related to neurodegenerative diseases in the future.
